# INITIAL – An observational study of disease severity in newly diagnosed asthma patients and initial response following 12 weeks’ treatment

**DOI:** 10.1038/s41598-018-36611-w

**Published:** 2019-02-04

**Authors:** Jiangtao Lin, Xiuhua Fu, Ping Jiang, Weidong Song, Xiaoyun Hu, Zhijun Jie, Chuntao Liu, Zhengguang He, Xiangdong Zhou, Huaping Tang

**Affiliations:** 1 Department of Pulmonary and Critical Care Medicine, China-Japan Friendship Hospital, Beijing, 100029 China; 2 Department of Pulmonary and Critical Care Medicine, The Affiliated Hospital of Inner Mongolia Medical University, Hohhot, China; 3 Department of Respiratory Diseases, Tianjin First Center Hospital, Tianjin, China; 4 Department of Respiratory Diseases, Peking University Shenzhen Hospital, Shenzhen, China; 5 Department of Respiratory Diseases, The First Affiliated Hospital of Shanxi Medical University, Shanxi, China; 6 Department of Respiratory Diseases, The Fifth People’s Hospital of Shanghai, Fudan University, Shanghai, China; 7 Department of Respiratory Diseases, West China Hospital, Sichuan University, Chengdu, China; 8 Department of Respiratory Diseases, Suining Central Hospital, Suining, China; 9 Department of Respiratory Diseases, Southwest Hospital, The First Affiliated Hospital of the Third Military Medical University, Chongqing, China; 10 Department of Respiratory Diseases, Qingdao Municipal Hospital, Qingdao, China

## Abstract

In China, there are an estimated 30 million people with asthma, a condition that remains poorly controlled in many patients. The INITIAL study (NCT02143739) was a 12-week, multicentre, prospective, observational study comprising 45 centres across Northern and Southern China that aimed to assess asthma severity among newly diagnosed patients as well as their prescribed medications and response to treatment. The primary objective was to evaluate asthma severity using Global Initiative for Asthma (GINA) 2006 research criteria. Secondary objectives included the distribution of asthma medication by GINA severity category and evaluation of GINA 2012-defined control levels. Medications were prescribed as per usual clinical practice. At baseline, among 4491 patients, 3.9%, 12.0%, 22.6% and 61.6% had intermittent, mild persistent, moderate persistent and severe persistent asthma, respectively. Inhaled corticosteroid/long-acting β_2_ agonist was the most common initial therapy in 90.2% of patients. GINA 2012-defined controlled asthma levels increased in all groups, rising from 6.1% at baseline to 43.0%, 53.8% and 67.8% at Weeks 4, 8 and 12, respectively. Most patients presented with severe persistent asthma. Newly diagnosed patients with asthma could benefit from at least 3 months of regular treatment followed by long-term pharmacological management.

## Introduction

In China, there are an estimated 30 million people with asthma^1^, a condition that remains poorly controlled in many patients^2–4^. A survey of 4125 outpatients with asthma (aged ≥17 years) conducted in mainland China using the Asthma Control Test (ACT)^5^ demonstrated that asthma was uncontrolled in 55.1% of patients (ACT score ≤19)^2^. A cross-sectional survey of 889 adult patients (aged ≥18 years) with moderate or severe asthma conducted in Jilin Province, China, demonstrated that asthma was partly controlled (ACT score 16–20) in 40.3% and controlled (ACT score 21–25) in 28.1% of patients at 1-year follow-up; asthma was unsatisfactorily controlled (ACT score ≤20) in 71.9% patients overall^3^. In Su *et al.’s* study of 2928 outpatients with asthma (aged ≥14 years) drawn from 10 major Chinese cities, using the Global Initiative for Asthma (GINA) 2006^6^ definition of symptom control (as judged by a physician), 26.2% had uncontrolled asthma over the preceding 12 months^4^. Of the remaining patients, 28.7% and 45.2% were judged to have controlled and partly controlled asthma, respectively^4^. Among the 402 Chinese patients with asthma surveyed during a wider Asia-Pacific study using the GINA definition of symptom control, 56% had partly controlled asthma and only 2% were judged to have controlled asthma^7^.

According to GINA, the long-term goal of asthma management is to achieve good symptom control and maintain normal activity levels while minimising the future risk of exacerbations, fixed airflow limitation and side effects of treatment^8^. Treatment with regular low-dose inhaled corticosteroids (ICS) is highly effective in reducing asthma symptoms and reducing the risk of asthma-related exacerbations, hospitalisation and death^8^. For patients at GINA Step 2 or above, ICS (with or without long-acting β_2_ agonists [LABA] depending on Step) is the recommended initial controller treatment^8^. GINA recommends that asthma severity be assessed retrospectively after a patient has been on controller treatment, with adjustment as necessary, over several months^8^. However, classification of asthma by severity may be useful for management decisions during initial assessments and to characterise patients not receiving ICS when initiating a study^6^. Furthermore, assessment of disease severity (intermittent, mild persistent, moderate persistent and severe persistent asthma) is recommended by the Chinese Guideline for Prevention and Management of Bronchial Asthma when selecting initial treatment^9,10^.

To date, few studies in China have addressed disease severity in newly diagnosed patients and their initial response to treatment. Therefore, this non-interventional study aimed to assess asthma severity among newly diagnosed Chinese patients who had not received ICS (the recommended initial controller treatment of choice in all but those with the mildest symptoms), their prescribed medications and response to treatment. In the context of this study, a new diagnosis of asthma was defined as one occurring no more than 3 months prior to enrolment. The primary objective was to evaluate asthma severity using GINA 2006 research criteria^6^ and the Chinese Thoracic Society Guidelines 2008^9^. The main secondary objectives were to assess the distribution of asthma medication by GINA severity category and evaluate GINA 2012-defined control levels during 12 weeks of treatment^11^.

## Results

### Patients

**Figure 1:**
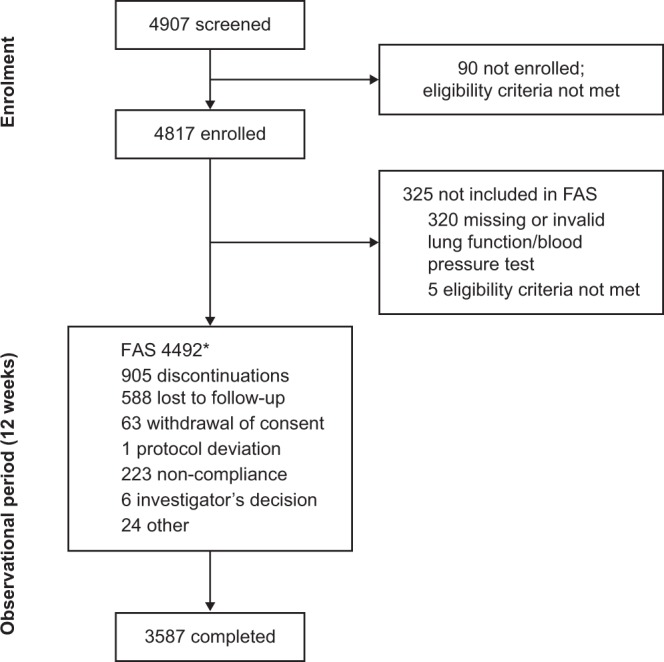
Patient flow. *One patient was considered to have completed the study as they were interviewed by telephone at Week 12 despite being considered discontinued by investigators. FAS, full analysis set.

Of the 4907 patients screened, 4817 were subsequently enrolled and 4492 were included in the full analysis set (FAS). Overall, 79.9% (3587) of the FAS completed the study (see Fig. 1 for patient flow). The majority of patients (92.8% [4168]) had no history of asthma. Of the 6.5% (293) patients with a history of asthma, the mean duration of illness was 10 days (range 1–83 days). Patient characteristics are given in Table 1.

**Table 1:** Patient characteristics.

		N = 4492
Age (year)*	<30, n (%)	850 (19.0)
	30–60, n (%)	3077 (68.8)
	>60, n (%)	543 (12.2)
Sex^†^	Male, n (%)	1819 (40.5)
	Female, n (%)	2672 (59.5)
Asthma history^†^	Yes, n (%)	293 (6.5)
	No, n (%)	4168 (92.8)
	Unknown, n (%)	30 (0.7)
BMI (kg/m^2^)^‡^	Mean (standard deviation)	23.9 (3.5)
	Range	15.2–42.5
Education level*	Illiterate, n (%)	145 (3.2)
	Primary school, n (%)	726 (16.2)
	Junior high school, n (%)	1261 (28.1)
	Technical secondary school or senior high school, n (%)	903 (20.1)
	Junior college or undergraduate, n (%)	1193 (26.6)
	University and above, n (%)	263 (5.9)
Occupation^§^	White-collar worker, n (%)	1572 (35.0)
	Blue-collar worker, n (%)	1340 (29.8)
	Student, n (%)	22 (2.7)
	Retired, n (%)	458 (10.2)
	Unemployed, n (%)	664 (14.8)
	Other, n (%)	335 (7.5)
Smoking status^†^	Never, n (%)	3381 (75.3)
	Ever, n (%)	635 (14.1)
	Current, n (%)	475 (10.6)
Area of residence^†^	Urban, n (%)	3208 (71.4)
	Rural, n (%)	1283 (28.6)
Insurance status^†^	Yes, n (%)	3972 (88.4)
	No, n (%)	519 (11.6)
Allergy history^†^	Yes, n (%)	992 (22.1)
	No, n (%)	2832 (63.1)
	Unknown, n (%)	667 (14.9)

^*^N = 4470; ^†^N = 4491; ^‡^N = 4489; ^§^N = 4391. BMI, body mass index.

### Asthma severity at baseline

According to GINA criteria, 173 (3.9%) patients had intermittent asthma, 538 (12.0%) patients had mild persistent asthma, 1013 (22.6%) patients had moderate persistent asthma and 2767 (61.6%) patients had severe persistent asthma (Table 2).

**Table 2:** Asthma severity at baseline.

		N = 4492, n (%)
Severity*	Intermittent	173 (3.9)
	Mild persistent	538 (12.0)
	Moderate persistent	1013 (22.6)
	Severe persistent	2767 (61.6)
Symptoms*	Less than once a week	673 (15.0)
	More than once a week but less than once a day	1749 (38.9)
	Daily	2069 (46.1)
Nocturnal symptoms*	Not more than twice a month	1256 (28.0)
	More than twice a month but less than once a week	793 (17.7)
	More than once a week	1198 (26.7)
	Frequent nocturnal asthma	1244 (27.7)
Exacerbations*	Brief exacerbations	1520 (33.9)
	Exacerbations may affect activity and sleep	1271 (28.3)
	Exacerbations affect activity and sleep	1461 (32.5)
	Frequent exacerbations	239 (5.3)
Daily use of SABA*	Yes	289 (6.4)
	No	4202 (93.6)
Limitation of physical activities*	Yes	1838 (40.9)
	No	2653 (59.1)

^*^N = 4491, one patient without baseline severity assessment was not included. SABA, short-acting β_2_ agonist.

### Medication prescribed at baseline

The most commonly prescribed baseline therapy was ICS/LABA (90.2%, 4051/4491), followed by leukotriene receptor antagonist (LTRA [62.1%, 2788/4491]), theophylline (14.3%, 643/4491), short-acting β_2_ agonist (SABA [11.4%, 512/4491]) and anti-cholinergic drugs (7.9%, 354/4491).

### Medication prescribed by baseline severity

For ICS/LABA, the most commonly prescribed initial therapy, the majority of patients were defined as moderate persistent (21.1%, 860/4049) and severe persistent (64.1%, 2595/4049) (Table 3).

**Table 3:** Initial medications prescribed by baseline severity.

Baseline severity	ICS/LABA, n (%)	ICS/LABA + LTRA, n (%)	LTRA without ICS/LABA, n (%)
Intermittent	129 (3.2)	73 (3.0)	31 (9.3)
Mild persistent	465 (11.5)	239 (9.7)	57 (17.2)
Moderate persistent	860 (21.2)	489 (19.9)	126 (38.0)
Severe persistent	2595 (64.1)	1654 (67.4)	118 (35.5)
Total*	4049 (100.0)	2455 (100.0)	332 (100.0)

^*^Missing data for two patients.

ICS, inhaled corticosteroid; LABA, long-acting β_2_ agonist; LTRA, leukotriene receptor antagonist.

ICS/LABA was prescribed to the majority of severe persistent patients at baseline, often in combination with LTRA. Patients with intermittent asthma represented 9.3% of those prescribed LTRA without ICS/LABA at baseline, greater than the proportion of those prescribed ICS/LABA alone or ICS/LABA in combination with LTRA (3.2% and 3.0%, respectively; Table 3). Several ICS/LABA formulations were prescribed at baseline; most patients received budesonide/formoterol (88.9%, 3602/4051), with only 10.2% (414/4051) receiving salmeterol/fluticasone and 0.6% (23/4051) receiving beclomethasone/formoterol. Severe persistent patients represented 57.5% of those prescribed salmeterol/fluticasone and 64.9% of those prescribed budesonide/formoterol (Table 4). There were more mild persistent patients prescribed salmeterol/fluticasone than budesonide/formoterol.

**Table 4:** Initial ICS/LABA type prescribed by baseline severity.

Baseline severity	Salmeterol/fluticasone, n (%)	Budesonide/formoterol, n (%)	Beclomethasone/formoterol, n (%)
Intermittent	8 (1.9)	120 (3.3)	0 (0.0)
Mild persistent	75 (18.1)	389 (10.8)	0 (0.0)
Moderate persistent	93 (22.5)	756 (21.0)	5 (21.7)
Severe persistent	238 (57.5)	2337 (64.9)	18 (78.3)
Total*	414 (100.0)	3602 (100.0)	23 (100.0)

^*^Missing data for 12 patients.

### Asthma control

**Figure 2:**
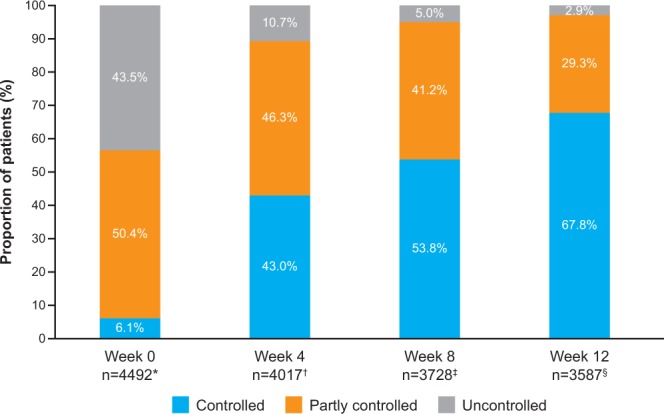
GINA 2012-defined asthma control levels. *Missing data for one patient; ^†^Missing data for 2942 patients; ^‡^Missing data for 2947 patients; ^§^Missing data for 964 patients.

Over the course of the study, the proportion of patients with GINA 2012-defined controlled asthma increased in all groups (Fig. 2), rising from 6.1% at baseline to 43.0%, 53.8% and 67.8% at Weeks 4, 8 and 12, respectively. The proportion of patients with GINA 2012-defined partly controlled and uncontrolled asthma decreased over the course of the study, with an overall change from baseline to Week 12 of 50.4% to 29.3% and 43.5% to 2.9%, respectively (Fig. 2). When asthma control was analysed by baseline severity, 61.6% of patients with severe persistent asthma were uncontrolled at baseline, falling to 4.2% at Week 12. At Week 12, among patients receiving budesonide/formoterol, the most common ICS/LABA combination, asthma was controlled in 67.2%, partly controlled in 29.7% and uncontrolled in 3.2%. The figures are similar to those observed in the FAS population at Week 12 irrespective of medicine type (67.8% controlled; 29.3% partly controlled; 2.9% uncontrolled).

### Asthma Control Questionnaire

Patient-reported asthma control was assessed using the Asthma Control Questionnaire (5-item version; ACQ-5)^12^. The ACQ-5 consists of five questions on symptom control scored on a scale of 0–6, in which mean scores of ≤0.75 indicate adequately controlled asthma and ≥1.5 indicate inadequately controlled asthma. An ACQ-5 score change of ±0.5 was deemed clinically important^13^. ACQ-5 scores indicated that asthma control improved over the course of the study (Table 5). At baseline, 15.2% of patients had adequately controlled asthma (ACQ-5 scores ≤0.75), rising to 80.6% at Week 12. Significant clinical improvement (ACQ score change ≥0.5) from baseline was seen in 71.8% (2881), 80.8% (3014) and 82.9% (2973) of patients at Weeks 4, 8 and 12, respectively (paired t-test, p < 0.0001).

**Table 5:** ACQ-5 scores.

ACQ-5 score	Week 0 (N = 4492)	Week 4 (N = 4017)	Week 8 (N = 3728)	Week 12 (N = 3587)
Mean (SD)	1.74 (1.00)	0.70 (0.78)	0.46 (0.67)	0.36 (0.61)
<0.75, n (%)	680 (15.2)	2454 (61.1)	2793 (74.9)	2891 (80.6)
0.75–1.5, n (%)	1308 (29.1)	977 (24.3)	632 (17.0)	473 (13.2)
>1.5, n (%)	2500 (55.7)	583 (14.5)	303 (8.1)	223 (6.2)
Total, n	4488	4014	3728	3587
Missing, n	4	3	0	0

ACQ-5, Asthma Control Questionnaire (5-item version); SD, standard deviation.

### Medication compliance

At Week 12, the majority (82.2%) of patients reported taking medication as prescribed by their physician at the previous visit. Missed doses were the most common form of non-compliance (16.4% of all patients).

### Risk factors associated with asthma control

Baseline severity, compliance, age, sex, occupation, education level, area of residence, allergy history, smoking status, standard of care at last visit and body mass index (BMI) reached significance (p < 0.15) in a univariate analysis of risk factors (Supplementary Table 1). Multivariate analysis showed that baseline severity, compliance, sex, occupation, education, insurance status and use of ICS and ICS combination at the last visit were significantly associated with better control at Week 12 (p < 0.05, Table 6).

**Table 6:** Multivariate analysis of risk factors associated with asthma control at Week 12.

Variable	Partly controlled vs. controlled		Uncontrolled vs. controlled	
	OR (95% CI)	p-value	OR (95% CI)	p-value
Baseline severity				
Intermittent vs. severe persistent	0.49 (0.22, 1.11)	0.087	0 (0, >9999)	0.980
** Mild persistent vs. severe persistent**	0.25 (0.15, 0.42)	<0.001	0 (0, >9999)	0.958
** Moderate persistent vs. severe persistent**	0.64 (0.44, 0.91)	0.014	0.1 (0.01, 0.77)	0.027
Compliance*				
** Good vs. poor**	0.67 (0.49, 0.92)	0.013	0.57 (0.22, 1.45)	0.238
Sex				
** Male vs. female**	1.73 (1.19, 2.5)	0.004	3.50 (1.22, 10)	0.020
Occupation				
** White-collar vs. blue-collar worker**	1.60 (1.02, 2.49)	0.040	0.20 (0.04, 0.95)	0.042
Student, retired, unemployed or others vs. blue-collar worker	1.20 (0.8, 1.8)	0.387	0.65 (0.22, 1.92)	0.433
Education				
Illiteracy vs. primary, junior high school, technical secondary school or senior high school	0.71 (0.26, 1.95)	0.510	1.73 (0.17, 18.04)	0.645
** Junior college or undergraduate, >B.S. degree vs. primary, junior high school, technical secondary school or senior high school**	0.62 (0.42, 0.91)	0.015	0.51 (0.12, 2.13)	0.358
Residence area				
Rural vs. urban	0.96 (0.65, 1.43)	0.858	0.28 (0.08, 1)	0.050
Insurance status				
** No vs. yes**	0.51 (0.3, 0.86)	0.013	0.71 (0.14, 3.52)	0.673
Allergy history				
No vs. yes	0.83 (0.62, 1.12)	0.233	0.53 (0.2, 1.42)	0.210
Smoking				
Ever vs. never	0.73 (0.44, 1.23)	0.235	1.13 (0.32, 3.98)	0.844
Current vs. never	0.77 (0.44, 1.32)	0.334	0.49 (0.09, 2.59)	0.401
Standard of care^†^		0.006		0.927
** ICS and ICS combination vs. non-SOC**	11.25 (2.57, 49.19)	0.001	0 (0, –)	0.994
ICS/LABA and combination vs. non-SOC	1.56 (0.82, 2.96)	0.173	1.53 (0.18, 13.13)	0.697
Age (year)	1.01 (1.00, 1.02)	0.060	1.01 (0.97, 1.06)	0.490
BMI	0.98 (0.94, 1.02)	0.243	1.02 (0.9, 1.16)	0.715

^*^Good compliance: During the observation period, the patient followed the doctor’s advice completely. ^†^Standard of care at last treatment. BMI, body mass index; B.S., bachelor of science; CI, confidence interval; ICS, inhaled corticosteroid; LABA, long-acting β_2_ agonist; OR, odds ratio; SOC, standard of care.

### Exacerbations

During the 12-week study, 96 patients (2.1%) had ≥1 exacerbation. Among 78 patients who required an emergency room (ER) visit, 55 were classified as severe persistent, 13 moderate persistent, seven mild persistent and three intermittent at baseline, respectively. The majority of patients requiring hospitalisation (12) were classified as severe persistent, and the remaining patients were moderate and mild persistent (one patient each). Patients requiring systemic corticosteroids for ≥3 days were distributed between severe persistent (eight) and moderate persistent (six) asthma. Of the severe persistent patients initially prescribed ICS/LABA, 2.5% experienced an exacerbation compared with 3.1% of those who were not.

## Discussion

In this non-interventional study of 4492 newly diagnosed patients with asthma who had not received prior therapy with ICS (the GINA-recommended initial controller therapy of choice at Step 2 or above), 61.6% presented with severe persistent asthma; 3.9%, 12.0% and 22.6% presented with intermittent, mild and moderate persistent asthma at baseline, respectively. A retrospective study of asthma trajectory over 10 years demonstrated that most patients classified as severe in the first year transitioned to less severe states in subsequent years^14^. While the severity measure used in that study was derived from medication use and markers of exacerbations^15^ rather than GINA criteria, it indicates that the course of severe asthma is potentially modifiable in line with the results presented here, albeit over a much shorter timescale.

At baseline, all but the intermittent group had low levels of asthma control. The proportion of patients with GINA 2012-defined controlled asthma increased in all severity groups, rising from 6.1% at baseline to 67.8% at Week 12. There was a corresponding decrease in the proportion of patients with partly controlled and uncontrolled asthma from baseline to Week 12 of 50.4% to 29.3% and 43.5% to 2.9%, respectively. Taken together, 97.1% of patients achieved GINA 2012-defined control or partial control at Week 12. These figures represent a marked improvement over those seen in the studies of Su *et al*. and Thompson *et al*., in which 73.8% and 58% of patients overall achieved GINA-defined control (28.7% and 2%) and partial control (45.2% and 56%), respectively^4,7^. Possible explanations for this discrepancy include differences in study design (post hoc survey vs. non-interventional study), patient population (asthma diagnosis ≥1 month vs. newly diagnosed) and treatment. Despite being the most commonly used regimen, less than half (45.6%) of patients surveyed by Su *et al*. were using ICS/LABA regularly (see below)^4^. A clinically significant improvement in ACQ-5 test score from baseline was seen in 72.8% patients at Week 4, rising to 82.9% patients at Week 12. This suggests that even 4 weeks of regular treatment can improve patient-reported asthma control. This improvement in patient-reported asthma control follows the trajectory of the observed increase in the proportion of patients with GINA-defined controlled and partly controlled asthma.

Overall, 90.2% of patients were initially prescribed ICS/LABA. This figure is considerably higher than the proportion of daily ICS/LABA use (45.6%) reported by Su *et al*.^4^. Possible explanations for this discrepancy include the fact that patients in that study were not newly diagnosed, having a mean duration of disease of 153 months^4^. Furthermore, it is unclear if they were visiting healthcare professionals (HCPs) as regularly as the patients in the current study. In the study by Yan *et al*., regular follow-up appointments were associated with a higher likelihood of continuing ICS/LABA or ICS/long-acting muscarinic antagonist (LAMA) for longer than 3 months^3^. At baseline, during the patients’ hospital stay, 92.2% were using ICS/LABA or ICS/LAMA daily; upon discharge, only 38.5% continued to do so for >3 months^3^. Results from the present study also showed no discernible difference in GINA 2012-derived control level at Week 12, irrespective of whether an LTRA was prescribed with an ICS/LABA at baseline.

In the present study, the level of GINA 2012-defined asthma control increased at each visit and was greater at Week 12 than Week 8, which may be related to treatment compliance. Multivariate analysis provided further evidence that treatment non-compliance is a risk factor for suboptimal asthma control, in line with the findings of the study by Zhong *et al*.^2^. Baseline severity, sex, occupation, education level and standard of care (ICS or ICS combination) at Week 12 were associated with good asthma control and should be subject to further investigation in the future.

As lung function testing is not mandatory as per the Chinese Thoracic Society Guidelines 2008^9^, the collection of lung function data after the initial visit was at the investigator’s discretion. This is a limitation of our study, as GINA 2012 control level could not be determined in 2942, 2947 and 964 patients at Weeks 4, 8 and 12, respectively. The GINA 2014 major revision altered the determination of symptom control by removing lung function testing from the assessment criteria^16^. Since all other criteria were unchanged in the revision and were collected during this study, a post hoc analysis of this data set using the latest GINA criteria (2018)^8^ is planned to assess how the level of asthma control compares with GINA 2012^11^. Control levels improved during the study, and the involvement in the trial itself combined with regular visits and contact with HCPs may have positively influenced the outcome. However, monthly visits do represent the standard of care in China.

In conclusion, over 60% of newly diagnosed patients in this large, observational study presented with severe persistent asthma and only 0.5% of these patients had controlled asthma at baseline. ICS/LABA was the most commonly prescribed initial therapy type, with most patients receiving budesonide/formoterol. Asthma control levels improved considerably following the introduction of therapy, and after 12 weeks of regular treatment two-thirds of patients achieved GINA 2012-defined controlled asthma. Greater baseline severity, medication non-compliance, male sex, educational level, occupation and non-standard care at Week 12 were identified as risk factors for poor asthma control. Asthma is a chronic disease; these results suggest that newly diagnosed patients with asthma could benefit from at least 3 months of regular treatment followed by long-term maintenance therapy. Further work is required, but this study provides evidence that special attention should be paid to those presenting with severe persistent asthma at baseline. Furthermore, patient education may be required to encourage patients to contact HCPs sooner and to maintain long-term therapy once symptoms improve.

## Methods

### Study design

The INITIAL study (NCT02143739) was a 12-week, multicentre, prospective, observational study comprising 45 tier 3 hospitals in major cities across Northern and Southern China. The study was conducted in accordance with the principles of the Declaration of Helsinki and Good Clinical Practice guidelines. Ethical approval was obtained from the ethics committee of the China-Japan Friendship Hospital, Beijing, China, the principal site, and from the local ethics committee at each site. Written consent was obtained from all patients. The first patient was enrolled on 7 June 2014 and the last patients completed the study on 13 September 2016. Patients visited the clinic four times over the 12-week (±7 days) study period.

### Patients

In this study, a new diagnosis of asthma was defined as one occurring no more than 3 months prior to enrolment. Patients newly diagnosed with asthma aged ≥18 years were eligible for study enrolment providing they were stable (i.e. no asthma exacerbation in the previous 2 weeks) and had not used ICS in the 3 months prior to enrolment. An exacerbation was defined as an asthma deterioration that required the use of systemic corticosteroids for ≥3 days, an ER visit or hospitalisation. Exacerbations that occurred within 14 days of each other were defined as one event. Patients who had participated in other clinical studies in the 3 months prior to enrolment or who had a diagnosis of/suspected chronic obstructive pulmonary disease were ineligible for enrolment.

### Assessments

At baseline, informed consent and medical history were obtained. Patients were screened and GINA-defined asthma severity^6^ and control were assessed^11^ as is standard clinical practice in China when initiating therapy^9,10^. Patient-reported outcomes were assessed using the ACQ-5^12^. Patients then visited the clinic every 4 weeks as per the usual clinical practice in China. GINA asthma control status, ACQ-5 and exacerbations were assessed at Weeks 4, 8 and 12. Treatment decisions were not part of the present study; medications, if any, were prescribed as per usual clinical practice at baseline, Week 4 and Week 8 with no additional monitoring or diagnostic procedures.

### Statistical analysis

The statistical analysis was primarily descriptive in nature. Quantitative variables were described by frequency, mean, standard deviation, median, minimum and maximum, and number of missing data. Qualitative variables were described using the absolute and relative (%) frequencies of each modality and number of missing data. Statistical tests were two-tailed and performed at the 0.05 significance level; 95% confidence intervals were calculated if applicable.

Changes in ACQ-5 test scores from baseline were analysed by paired t-test. Factors influencing asthma control status at Week 12 were investigated using regression analysis. Variables with a p-value ≤ 0.15 in univariate analysis were subsequently included in multivariate analysis.

### Study size

According to unpublished market research conducted in 2010, among 800 newly diagnosed asthma patients in China, the proportion of patients with moderate and severe asthma was approximately 89%. Based on this assumption, with a ~30% drop-out rate, a sample size of approximately 5000 patients will provide a 95% confidence level with a 0.9% margin of error.

## Electronic supplementary material

Supplementary Information

## Data Availability

The datasets generated and/or analysed during the current study are available via the AstraZeneca Group of Companies – Data Request Portal at: https://astrazenecagroup-dt.pharmacm.com/DT/Home. More information on AstraZeneca’s clinical trials disclosure policy is available at: http://astrazenecagrouptrials.pharmacm.com//ST/Submission/Disclosure.

## References

[1] Lin J (2013). Review and outlook of asthma management within the past 60 years in China. Zhonghua Jie He He Hu Xi Za Zhi.

[2] Zhong N (2016). Uncontrolled asthma and its risk factors in adult Chinese asthma patients. Ther. Adv. Respir. Dis..

[3] Yan B (2016). Asthma control and severe exacerbations in patients with moderate or severe asthma in Jilin Province, China: a multicenter cross-sectional survey. BMC Pulm. Med..

[4] Su N (2014). Evaluation of asthma control: a questionnaire-based survey in China. Chin. Med. J..

[5] Schatz M (2006). Asthma Control Test: reliability, validity, and responsiveness in patients not previously followed by asthma specialists. J. Allergy Clin. Immunol..

[6] Global Initiative for Asthma (GINA). GINA report: global strategy for asthma management and prevention (2006).

[7] Thompson PJ (2013). Insights, attitudes and perceptions about asthma and its treatment: findings from a multinational survey of patients from 8 Asia-Pacific countries and Hong Kong. Respirology.

[8] Global Initiative for Asthma (GINA). GINA report: global strategy for asthma management and prevention (2018).

[9] Chinese Thoracic Society (2008). Guideline for management of asthma (definition, diagnosis, treatment and management of asthma) from Asthma Study Group of Chinese Thoracic Society. Zhonghua Jie He He Hu Xi Za Zhi.

[10] Asthma Workgroup, Chinese Thoracic Society & Chinese Medical Association (2014). Chinese expert consensus on bronchial asthma control. J. Thorac. Dis..

[11] Global Initiative for Asthma (GINA). GINA report: global strategy for asthma management and prevention (2012).

[12] Juniper EF, O’Byrne PM, Roberts JN (2001). Measuring asthma control in group studies: do we need airway calibre and rescue beta2-agonist use?. Respir. Med..

[13] Juniper EF, Svensson K, Mork AC, Stahl E (2005). Measurement properties and interpretation of three shortened versions of the asthma control questionnaire. Respir. Med..

[14] Chen W (2016). The natural history of severe asthma and influences of early risk factors: a population-based cohort study. Thorax.

[15] Firoozi F, Lemière C, Beauchesne M, Forget A, Blais L (2007). Development and validation of database indexes of asthma severity and control. Thorax.

[16] Global Initiative for Asthma (GINA). GINA report: global strategy for asthma management and prevention (2014).

