# Supplementary material for: INITIAL – An observational study of disease severity in newly diagnosed asthma patients and initial response following 12 weeks’ treatment
Source: Sci Rep. 2019 Feb 4;9:1254. doi: 10.1038/s41598-018-36611-w (PMC6362102; doi:10.1038/s41598-018-36611-w)
Supplement: Supplementary file 1 — Supplementary Information [file 41598_2018_36611_MOESM1_ESM.docx]

# INITIAL – An observational study of disease severity in newly diagnosed asthma patients and initial response following 12 weeks’ treatment

## Authors

Jiangtao Lin,^1*^ Xiuhua Fu,^2^ Ping Jiang,^3^ Weidong Song,^4^ Xiaoyun Hu,^5^ Zhijun Jie,^6^ Chuntao Liu,^7^ Zhengguang He,^8^ Xiangdong Zhou,^9^ Huaping Tang^10^

1. Department of Pulmonary and Critical Care Medicine, China-Japan Friendship Hospital, Beijing 100029, China

2. Department of Pulmonary and Critical Care Medicine, The Affiliated Hospital of Inner Mongolia Medical University, Hohhot, China

3. Department of Respiratory Diseases, Tianjin First Center Hospital, Tianjin, China

4. Department of Respiratory Diseases, Peking University Shenzhen Hospital, Shenzhen, China

5. Department of Respiratory Diseases, The First Affiliated Hospital of Shanxi Medical University, Shanxi, China

6. Department of Respiratory Diseases, The Fifth People's Hospital of Shanghai, Fudan University, Shanghai, China

7. Department of Respiratory Diseases, West China Hospital, Sichuan University, Chengdu, China

8. Department of Respiratory Diseases, Suining Central Hospital, Suining, China

9. Department of Respiratory Diseases, Southwest Hospital, The First Affiliated Hospital of the Third Military Medical University, Chongqing, China

10. Department of Respiratory Diseases, Qingdao Municipal Hospital, Qingdao, China

* Corresponding author: Jiangtao Lin, Department of Pulmonary and Critical Care Medicine, China-Japan Friendship Hospital, No.2 Yinghua East Street, Chaoyang District, Beijing 100029, China. Tel: 86-10-84206187; Fax: 86-10-64200588; E-mail: jiangtao_l@263.net

**Supplementary Table 1. Univariate analysis of risk factors for control status at Week 12**

| **Parameter** | **Controlled at Week 12 (N=1778)** | **Partly controlled at Week 12 (N=768)** | **Uncontrolled at Week 12 (N=77)** | **p-value** |
| --- | --- | --- | --- | --- |
| Baseline severity categories |  |  |  | <0.0001 |
| Intermittent, n (%) | 96 (5.40) | 18 (2.34) | 0 (0.00) |  |
| Mild persistent, n (%) | 272 (15.30) | 50 (6.51) | 2 (2.60) |  |
| Moderate persistent, n (%) | 448 (25.20) | 139 (18.10) | 9 (11.69) |  |
| Severe persistent, n (%) | 962 (54.11) | 561 (73.05) | 66 (85.71) |  |
| Total, n | 1778 | 768 | 77 |  |
| Compliance* |  |  |  | <0.0001 |
| Poor, n (%) | 450 (25.31) | 269 (35.03) | 31 (40.26) |  |
| Good, n (%) | 1328 (74.69) | 499 (64.97) | 46 (59.74) |  |
| Total, n | 1778 | 768 | 77 |  |
| Age (year) |  |  |  | <0.0001 |
| N (missing) | 1770 (8) | 766 (2) | 77 (0) |  |
| Mean (SD) | 42.18 (13.14) | 44.99 (13.13) | 47.05 (12.33) |  |
| Min, max | 18, 89 | 18, 81 | 20, 72 |  |
| Md (Q3–Q1) | 42.00 (20.00) | 45.00 (22.00) | 48.00 (17.00) |  |
| Q1, Q3 | 31.00, 51.00 | 34.00, 56.00 | 40.00, 57.00 |  |
| Age (year) |  |  |  | 0.0003 |
| <30, n (%) | 368 (20.79) | 113 (14.75) | 10 (12.99) |  |
| 30–60, n (%) | 1205 (68.08) | 535 (69.84) | 54 (70.13) |  |
| ≥60, n (%) | 197 (11.13) | 118 (15.40) | 13 (16.88) |  |
| Total, n | 1770 | 766 | 77 |  |
| Missing, n | 8 | 2 | 0 |  |
| Sex |  |  |  | 0.0005 |
| Male, n (%) | 658 (37.01) | 340 (44.27) | 38 (49.35) |  |
| Female, n (%) | 1120 (62.99) | 428 (55.73) | 39 (50.65) |  |
| Total, n | 1778 | 768 | 77 |  |
| Race |  |  |  | 0.5962 |
| White, n (%) | 0 (0.00) | 0 (0.00) | 0 (0.00) |  |
| Black, n (%) | 3 (0.17) | 0 (0.00) | 0 (0.00) |  |
| Asian, n (%) | 1775 (99.83) | 768 (100.00) | 77 (100.00) |  |
| Others, n (%) | 0 (0.00) | 0 (0.00) | 0 (0.00) |  |
| Total, n | 1778 | 768 | 77 |  |
| Occupation |  |  |  | 0.0046 |
| White-collar worker, n (%) | 666 (37.46) | 263 (34.24) | 13 (16.88) |  |
| Blue-collar worker, n (%) | 532 (29.92) | 240 (31.25) | 32 (41.56) |  |
| Student, retired, unemployed or 　　other, n (%) | 580 (32.62) | 265 (34.51) | 32 (41.56) |  |
| Total, n | 1778 | 768 | 77 |  |
| Education |  |  |  | <0.0001 |
| Illiteracy, n (%) | 53 (2.98) | 23 (2.99) | 1 (1.30) |  |
| Primary, junior high school, technical 　　secondary school or senior high 　　school, n (%) | 1101 (61.92) | 530 (69.01) | 64 (83.12) |  |
| Junior college, undergraduate, >B.S. 　　degree, n (%) | 624 (35.10) | 215 (27.99) | 12 (15.58) |  |
| Total, n | 1778 | 768 | 77 |  |
| Residence area |  |  |  | 0.0364 |
| Urban, n (%) | 1320 (74.24) | 544 (70.83) | 49 (63.64) |  |
| Rural, n (%) | 458 (25.76) | 224 (29.17) | 28 (36.36) |  |
| Total, n | 1778 | 768 | 77 |  |
| Insurance status |  |  |  | 0.0133 |
| Yes, n (%) | 1564 (87.96) | 703 (91.54) | 72 (93.51) |  |
| No, n (%) | 214 (12.04) | 65 (8.46) | 5 (6.49) |  |
| Total, n | 1778 | 768 | 77 |  |
|  |  |  |  |  |
| Family history of asthma |  |  |  | 0.1924 |
| Yes, n (%) | 354 (19.91) | 185 (24.09) | 18 (23.38) |  |
| No, n (%) | 1353 (76.10) | 552 (71.88) | 56 (72.73) |  |
| Unknown, n (%) | 71 (3.99) | 31 (4.04) | 3 (3.90) |  |
| Total, n | 1778 | 768 | 77 |  |
| Allergy history |  |  |  | 0.1430 |
| Yes, n (%) | 385 (21.65) | 186 (24.22) | 16 (20.78) |  |
| No, n (%) | 1087 (61.14) | 467 (60.81) | 54 (70.13) |  |
| Unknown, n (%) | 306 (17.21) | 115 (14.97) | 7 (9.09) |  |
| Total, n | 1778 | 768 | 77 |  |
| Smoking |  |  |  | 0.0007 |
| Never, n (%) | 1394 (78.40) | 550 (71.61) | 51 (66.23) |  |
| Ever, n (%) | 210 (11.81) | 128 (16.67) | 16 (20.78) |  |
| Current, n (%) | 174 (9.79) | 90 (11.72) | 10 (12.99) |  |
| Total, n | 1778 | 768 | 77 |  |
| Smoking index^†^ |  |  |  | 0.0002 |
| N (missing) | 383 (1) | 217 (1) | 26 (0) |  |
| Mean (SD) | 14.52 (16.53) | 17.90 (16.73) | 22.35 (14.91) |  |
| Min, max | 0.025, 96 | 0.05, 120 | 1.5, 60 |  |
| Md (Q3–Q1) | 10.00 (17.50) | 15.00 (25.00) | 20.00 (20.00) |  |
| Q1, Q3 | 2.50, 20.00 | 5.00, 30.00 | 10.00, 30.00 |  |
| BMI |  |  |  | 0.1141 |
| N (missing) | 1778 (0) | 768 (0) | 77 (0) |  |
| Mean (SD) | 23.82 (3.54) | 23.98 (3.65) | 24.70 (3.55) |  |
| Min, max | 15.79, 42.52 | 16, 38.2 | 17.9, 34.37 |  |
| Md (Q3–Q1) | 23.53 (4.82) | 23.78 (5.03) | 23.95 (3.88) |  |
| Q1, Q3 | 21.22, 26.04 | 21.28, 26.31 | 22.52, 26.40 |  |
| Standard of care^‡^ |  |  |  | 0.0009 |
| ICS and ICS combination, n (%) | 21 (1.18) | 19 (2.48) | 1 (1.30) |  |
| ICS/LABA and combination, n (%) | 1586 (89.45) | 704 (91.79) | 74 (96.10) |  |
| Non-SOC, n (%) | 166 (9.36) | 44 (5.74) | 2 (2.60) |  |
| Total, n | 1773 | 767 | 77 |  |
| Missing, n | 5 | 1 | 0 |  |

* Good compliance: During the observation period, the patient followed the doctor’s advice completely

^†^ Smoking index contains both ever and current smoking patients

^‡^ Standard of care at last treatment

BMI, body mass index; B.S., bachelor of science; ICS, inhaled corticosteroid; LABA, long-acting β_2_ agonist; Md, median; Q, quartile; SD, standard deviation; SOC, standard of care
